# Clinical-epidemiological assessment of patients undergoing bariatric and metabolic surgery in a medium-complexity service in Maranhão, Brazil

**DOI:** 10.1590/0100-6991e-20243708-en

**Published:** 2024-07-05

**Authors:** LIVIO MELO BARBOSA, BRUNA PEREIRA CARVALHO SIRQUEIRA, JOSÉ THIAGO OLIVEIRA DE CARVALHO, ALBERTO NÉLIO BANDEIRA BARROS, ANDERSON BENTES DE LIMA

**Affiliations:** 1 - Universidade Federal do Maranhão, Curso de Medicina - Centro de Ciências de Imperatriz - Imperatriz - MA - Brasil; 2 - Clínica de Saúde Nutrogastro - Centro De Cirurgia Geral, Digestiva e Obesidade - Imperatriz - MA - Brasil; 3 - Universidade Estadual do Pará - Programa de Mestrado Profissional em Cirurgia e Pesquisa Experimental - Centro de Ciências da Saúde - Belém - PA - Brasil

**Keywords:** Bariatric Surgery, Obesity, Comorbidity, Prevalence, Epidemiology, Cirurgia Bariátrica, Obesidade, Comorbidade, Prevalência, Epidemiologia

## Abstract

**Introduction:**

the obesity is defined as the excessive accumulation of fat in different areas of the body, a condition that causes damage to health and is a critical risk factor for various comorbidities. Bariatric surgery is the therapeutic option with the best results.

**Methods:**

this is a retrospective descriptive study using data obtained from medical records from January 2018 to December 2020 on patients undergoing bariatric surgery. Statistical analysis used a significance level of p<0.05.

**Results:**

178 medical records were included, 77.5% of which were women. The average age was 35.7 years (± 9.5), 63.8% of the patients were from Imperatriz, 98.3% reported a sedentary lifestyle, 38.7% regular alcohol consumption and 13% smoking. The prevalence of Class III obesity (BMI≥40 kg/m²) was 53.3%. The most common comorbidities were hepatic steatosis (64.6%), type 2 diabetes mellitus (DM2) (40.5%) and hypertension (38.7%). The main type of surgery performed was Roux-en-Y gastric bypass (RYGB) (89.3%). There was an association between median BMI and gender (p=0.008), with women showing higher values [43.4 (IQR 39.1 - 48.8)]. The mean BMI of patients who underwent RYGB was significantly higher compared to those who underwent vertical gastrectomy (VG) (p=0.009). There was a statistical association between DM2 (p=0.033) and depression (p=0.018) and the type of surgery performed.

**Conclusion:**

the clinical and epidemiological profile found showed a higher prevalence of females and individuals with Class III obesity. RYGB was the most commonly performed procedure, establishing an association with BMI and some of the patients’ comorbidities.

## RESUMO

**Introdução:**

a obesidade é definida pelo acúmulo excessivo de gordura em diferentes regiões corporais, condição que acarreta prejuízos à saúde e constitui fator de risco para diversas comorbidades. A cirurgia bariátrica é a opção terapêutica com melhores resultados para o seu tratamento.

**Métodos:**

estudo retrospectivo descritivo realizado com dados obtidos de prontuários médicos do período de janeiro/2018 a dezembro/2020, relacionados a pacientes submetidos à cirurgia bariátrica. As análises estatísticas realizadas adotaram nível de significância p<0,05.

**Resultados:**

foram incluídos 178 prontuários, sendo 77,5% de mulheres. A média de idade foi de 35,7 anos (± 9,5), 63,8% dos pacientes eram procedentes de Imperatriz, 98,3% relataram sedentarismo, 38,7% consumo regular de álcool e 13% tabagismo. A prevalência de obesidade grau III (IMC≥40 kg/m²) foi de 53,3%. As comorbidades mais relacionadas foram esteatose hepática (64,6%), diabetes mellitus tipo 2 (DM2) (40,5%) e hipertensão arterial (38,7%). O principal tipo de cirurgia realizada foi o by-pass gástrico em Y de Roux (BGYR) (89,3%). Observou-se associação entre a mediana de IMC e o sexo (p=0,008), com as mulheres apresentando maiores valores [43,4 (IIQ 39,1 - 48,8)]. A média de IMC dos pacientes submetidos ao BGYR foi significativamente maior comparado aos que realizaram gastrectomia vertical (GV) (p=0,009). Houve associação estatística entre o DM2 (p=0,033) e a depressão (p=0,018) com o tipo de cirurgia realizada.

**Conclusão:**

o perfil clínico-epidemiológico encontrado evidenciou maior prevalência do sexo feminino e de indivíduos com obesidade grau III. O BGYR foi o procedimento mais realizado, estabelecendo associação com IMC e algumas comorbidades apresentadas pelos pacientes.

## INTRODUCTION

The World Health Organization (WHO) defines obesity as the excessive or abnormal accumulation of body fat, which causes damage to health and constitutes a critical risk factor for several comorbidities^1^ ^,^ ^2^. According to 2020 data from the Brazilian Institute of Geography and Statistics (IBGE), obesity among adults in the country increased from 11.8% to 20.6% between 2006 and 2019, and overweight affects more than 55% of these individuals^3^ ^-^ ^5^.

The management of obesity is multidisciplinary and rigorous. Although lifestyle changes and pharmacological treatment contribute to weight loss and control of comorbidities, most patients have difficulties in obtaining good long-term results^6^. In this context, bariatric and metabolic surgery is the therapeutic option with the greatest benefits for the management of morbidity and metabolic conditions associated with weight^7^ ^-^ ^9^. Its methods have been innovated over the years, ensuring an increasingly significant weight loss and control of metabolic parameters^10^ ^,^ ^11^. 

Every year the number of bariatric and metabolic surgeries grows. According to data from the Brazilian Society of Bariatric and Metabolic Surgery (SBCBM), in 2019 more than 68,500 procedures were performed, of which about 56,000 in the private sector and 12,500 in the public network. However, these data do not reflect the total number of operations in the country, as there is no national database^11^ ^,^ ^12^. 

In the state of Maranhão, only the University Hospital of the Federal University of Maranhão (HU-UFMA), located in the city of São Luís, is accredited by the Ministry of Health and can perform this procedure free of charge^13^. In the city of Imperatriz, the second largest in Maranhão and the main center of the state’s Southern Health Macro-region, which assists about 1.2 million individuals and serves as an interstate reference for the states of Tocantins and Pará, this service is exclusively private.

Although increasing, access to bariatric and metabolic surgery varies in the localities and little is known about the trend of the different profiles of patients undergoing it, especially in regions with reduced accessibility to the service. Access to the information is necessary to assess the quality of care and obtain data on the long-term outcomes of the procedure. 

In this context, this research aimed to determine the clinical and epidemiological profile of patients who underwent bariatric and metabolic surgery in a medium-complexity service in Imperatriz, MA.

## METHODS

This is an observational, retrospective, and descriptive study involving patients in two medium-complexity services in the city of Imperatriz, state of Maranhão, Brazil, the Clínica Diagnóstica and the Clínica de Saúde Nutrogastro, references for the surgical treatment of obesity in Southern Maranhão, as well as in cities in the adjacent states of Tocantins and Pará. To this end, we used data obtained from the medical records of patients who underwent bariatric and metabolic surgery in the institutions, from January 2018 to December 2020.

The sample consisted of 180 medical records, corresponding to the number of patients treated during the period evaluated. Of these, two did not meet the study criteria. Therefore, the final analysis was based on data from 178 patients. The inclusion and exclusion criteria are described in Table 1.

**Table 1:** Inclusion and exclusion criteria for the study.

Inclusion criteria	Exclusion Criteria
Patients aged ≥ 18 years, of both sexes, who underwent bariatric surgery between January 2018 and December 2020.	Patients with medical records with insufficient information to fill the research form.

Source: authors (2024).

For data collection, the authors prepared a form based on the literature, which was filled based on the information contained in the medical records. The data analyzed were as follows: Personal data: sex, age, marital status, and ethnicity; Clinical data: body mass index (BMI), degree of obesity, lifestyle habits (sedentary lifestyle, alcoholism, and smoking), associated comorbidities (hepatic steatosis, systemic arterial hypertension (SAH), diabetes mellitus (DM), dyslipidemia, sleep apnea, depression, osteoarthrosis, gastroesophageal reflux disease (GERD), and the type of surgery performed (gastric bypass or sleeve gastrectomy)).

The data obtained were stored in Microsoft® Office Excel 2016 software. Subsequently, they were imported into the open-source software R Studio (R Core Team, 2022) for statistical analysis. Subsequently, they were organized in tables and presented in absolute and relative numbers. To compare the proportions between the groups, we used the Student’s t-test and the Mann-Whitney U test based on the variables’ distribution pattern, and we expressed the results as mean and standard deviation or median and 25%-75% interquartile range (IQR). To evaluate quantitative variables, we applied the Fisher’s exact test. The level of significance was set at 0.05.

All data were collected in an exclusive room and access to medical records was restricted to researchers, who previously committed to the confidentiality of the information by signing the Data Use Commitment Term. The research was approved by the Human Research Ethics Committee of the University Hospital of the Federal University of Maranhão (HU-UFMA), under protocol number CAAE 56081821.0.0000.5086, opinion number: 5.314.867.

## RESULTS

The analysis included 178 patients who underwent bariatric surgery from January 2018 to December 2020, of whom 77.5% (138) were female and 22.5% (40), male. The mean age was 35.7 years (± 9.5), ranging from 18 to 67.

Regarding marital status, 57.8% reported being married, 32% single, and 10.2% widowed. Of the total, 63.8% came from the municipality of Imperatriz and the others from other municipalities in the interior of Maranhão, Tocantins, and Pará, such as Balsas (MA), Porto Franco (MA), Açailândia (MA), Bom Jesus (TO), Rondon (PA), and Parauapebas (PA), among others. In addition, 98.3% reported a sedentary lifestyle, 13% smoked, and 38.7% reported frequent alcohol consumption. Table 2 shows patients’ epidemiological profile.

**Table 2:** Epidemiological profile of patients undergoing bariatric surgery.

Variables	n	%
Sex		
Male	40	22,5
Female	138	77,5
Marital status		
Single	57	32
Married	103	57,8
Divorced	18	10,2
Widowed	0	0
Origin		
Imperatrz	111	63,8
Another city	63	36,2
Sedentary lifestyle		
Yes	175	98,3
No	3	1,7
Variables	n	%
Alcoholism		
Yes	69	38,7
No	109	61,3
Smoking		
Yes	23	13
No	155	87

Source: authors (2024). Data presented as absolute (n) and relative (%) values.

All evaluated individuals reported some comorbidity. We observed hepatic steatosis in 64.6%, type-2 diabetes mellitus (T2DM) in 40.5%, (SAH) in 38.7%, and GERD in 28.7%, these being the most frequent conditions. Regarding the classification of obesity, 3.4% had grade I obesity, 43.3% grade II, and 53.3% grade III obesity, based on the Body Mass Index (BMI), as described in Table 3. The median BMI was 40.2kg/m² (IQR 37.2-44), ranging from 33.0kg/m² to 60.7kg/m².

**Table 3:** Prevalence of comorbidities and type of surgery performed.

Variables	n	%
Degree of obesity		
Grade I	6	3,4
Grade II	77	43,3
Grade III	95	53,3
Systemic Arterial Hypertension		
Yes	69	38,7
No	109	61,3
Type-II Diabetes Mellitus		
Yes	72	40,5
No	106	59,5
Dyslipidemia		
Yes	45	25,3
No	133	74,7
Sleep Apnea		
Yes	3	1,7
No	175	98,3
Depression		
Yes	6	3,4
No	172	96,6
Hepatic Steatosis		
Yes	115	64,6
No	63	35,4
Variables	n	%
GERD		
Yes	51	28,7
No	127	71,3
Osteoarthritis		
Yes	42	23,6
No	136	76,4
RYGB^╬^	159	89,3
SG^╬╬^	19	10,7

RYGB: Roux-en-Y gastric bypass; SG: Sleeve Gastrectomy. Data presented as absolute (n) and relative (%) values.

Regarding the type of surgery performed, 89.3% of the patients underwent Roux-en-Y gastric bypass (RYGB), and 10.7%, sleeve gastrectomy (SG). When analyzing the differences between the mean BMI and the types of surgeries, we found a significant relationship (p=0.009), with the patients who underwent RYGB having higher mean BMI, as shown in Table 4.

**Table 4:** Difference between means and medians between types of surgeries.

Variables	SG	RYGB	p-value
Body Mass Index*	38,0 (3,38)	40,6 (5,37)	0,009
Age**	34,0 (18,0 - 32,5)	38,0 (35,0 - 42,0)	0,182

Source: authors (2024). SG: Sleeve Gastrectomy; RYGB: Roux-en-Y gastric bypass; *Student’s t-test (normal distribution of the variable, represented by mean and standard deviation); **Mann-Whitney (non-normal distribution of the variable, represented by median and interquartile ranges).

When verifying possible associations between the analyzed variables and BMI, we found statistical significance for sex, women having higher median BMI than men, 43.3kg/m² (39.1-48.8) versus 39.9kg/m² (37-42.9), respectively (p=0.008). We could not find a statistically significant relationship when relating this parameter to the comorbidities analyzed, as can be seen in Table 5.

**Table 5:** BMI and categorical variables.

	Median BMI	IQR	p-value*
Sex			
Male	39,9	37,0 - 42,9	0,008
Female	43,4	39,1 - 48,8	
Systemic Arterial Hypertension			
Yes	40,4	37,1 - 45,7	0,399
No	40	37,4 - 43,0	
Type-2 Diabetes Mellitus			
Yes	39,9	37,5 - 43,9	0,82
No	40,4	37,1 - 44,0	
Dyslipidemia			
Yes	40	37,6 - 43,6	0,93
No	40,4	37,1 - 44,0	
Depression			
Yes	37,8	36,4 - 41,9	0,476
No	40,2	37,2 - 44,0	
Hepatic Steatosis			
Yes	40,4	36,6 - 44,0	0,813
No	40	37,5 - 43,8	
GERD			
Variables	n	%	p-valor
Yes	40,6	37,9 - 43,8	0,281
No	40	37,1 - 43,9	
Osteoarthritis			
Yes	40,1	37,5 - 44,0	0,364
No	40,1	36,2 - 43,1	

Source: authors (2024). *Mann-Whitney test (non-normal distribution of variables).

We observed statistically relevant relationships (p<0.05) between the degree of patients’ obesity with the type of bariatric and metabolic surgery performed, so that most patients underwent RYGB, especially those with grade III obesity and with T2DM, depression, and osteoarthrosis. All other variables did not show significant relevance when associated with the type of surgical technique, as shown in Table 6. 

**Table 6:** Clinical profile of patients undergoing RYGB and SG.

Variable	RYGB		SG		p-value*
	n	%	n	%	
Sex					
Male	39	25,0	1	25,0	
Female	117	94,7	18	5,3	0,079
Degree of obesity					
Grade I	4	2,5	1	5,3	0,031
Grade II	62	39	13	68,4	
Grade III	95	58,5	5	26,3	
Systemic Arterial Hypertension					
Yes	63	40,4	6	31,6	0,622
No	93	59,6	13	68,4	
Type-2 Diabetes Mellitus					
Yes	69	44,2	3	15,8	0,033
No	87	55,8	16	84,2	
Dyslipidemia					
Yes	41	26,3	4	16,1	0,830
No	115	73,7	15	78,9	
Depression					
Yes	3	1,9	3	15,8	0,018
No	153	98,1	16	84,2	
Osteoarthritis					
Yes	41	26,3	1	5,3	0,047
No	115	73,7	18	94,7	
Hepatic Steatosis					
Yes	104	66,7	9	47,4	0,160
No	52	33,3	10	52,6	
GERD					
Yes	46	29,5	3	15,8	0,325
No	110	70,5	16	84,2	

Source: authors (2024). *Fisher’s exact test (proportions were ≤ 5%). Data presented as absolute (n) and relative (%) values. RYGB: Roux-en-Y gastric bypass; SG: Sleeve Gastrectomy.

## DISCUSSION

From 2018 to 2020, 178 bariatric and metabolic surgeries were performed in the city of Imperatriz - MA, with a predominance of grade III obesity (53.3%) associated with hepatic steatosis, T2DM, and SAH. 

From this perspective, 77.5% of the patients undergoing the procedure were female, a fact also identified in other similar studies. According to IBGE data, in 2019, obesity among women in the country increased from 14.5% to 30.2%, this condition being more prevalent in women^5^.

According to Ribeiro et al.^14^, the high stress to which women are exposed because of the double shift of work and domestic care makes them more likely to develop weight-related disorders, such as overweight and obesity. Moreover, the better perception of health and self-care, in addition to the concern for aesthetics, may also justify the greater search for the procedure by women^15^ ^,^ ^16^. 

Regarding the age group, most operated individuals had a mean age of 35.7 (±9.5) years, similar to the findings of Palheta et al.^17^ and Junges et al.^18^. According to national data, in Brazil the age group between 35 and 44 years old was the one with the highest obesity rate between 2006 and 2018, with a prevalence of 84.2%^5^ ^,^ ^19^. 

Lifestyle is a determining aspect for health and the development of obesity^3^ ^,^ ^20^. Most studied patients reported a sedentary lifestyle (98.3%), smoking (13%), and alcohol consumption (38.7%). In Brazil, in 2016, approximately 50% of the adult population did not exercise regularly^21^. On the other hand, Silva et al.^22^ examined the profile of preoperative patients for bariatric surgery in Santa Maria, Rio Grande do Sul, Brazil, and found a rate of 69% of regular physical activity. This suggests greater awareness among individuals in the region or, possibly, more effective care provided by the Health Service. 

Regarding tobacco and alcohol consumption, the literature shows an association with overweight and obesity, considering that these substances are capable of altering several organic pathways due to their toxic potential, influencing body weight levels^23^ ^-^ ^25^. The data found corroborate the studies by Silva et al.^22^ and King et al.^26^, with a prevalence of smoking (13%) and alcohol consumption (14.8%), respectively. 

Obesity as a predictor of morbidity and mortality has several definitions, with BMI being the most used metric for anthropometric classification and an important criterion for indication of bariatric surgery^27^ ^,^ ^28^. Studies have shown that cardiovascular risk almost doubles in individuals with grade III obesity compared with those with grade II^29^. In this study, more than half of the patients had a BMI ≥40kg/m² (53.3%), which is the main indication for the procedure. The results agree with those found by Arantes et al.^30^ and Silva et al.^22^, who also identified this prevalence.

Regarding comorbidities, the most prevalent were hepatic steatosis (64.6%), T2DM (40.5%), and hypertension (38.7%). In addition, patients with higher BMI had a higher number of these associated health conditions. Arantes et al.^30^ developed a study with a similar approach, finding that the main associated diseases were T2DM (89%), hypertension (74.8%), and hepatic steatosis (68.2%). 

In this scenario, with a high BMI (grade III) associated with comorbidities, the therapy with the best results is bariatric surgery. The literature indicates that the most performed techniques are gastric bypass and sleeve gastrectomy^37^ ^,^ ^38^. In this study, 89.3% of the performed procedures were RYGB and 10.7% were SG. 

Currently, SG is the most performed bariatric and metabolic surgery technique in the world, as it is simpler and requires less operative time, representing more than 60% of all procedures^38^. Despite this, a multicenter randomized clinical trial revealed greater weight loss in patients undergoing RYGB compared with SG, despite having a higher risk of complications, a factor that influences the choice of the most appropriate technique for each patient^39,40^. According to surveys, in Brazil RYGB is the leader, corresponding to almost two-thirds of all these surgeries in the country, corroborating the findings of this study^38^. 

In this regard, in addition to the treatment of obesity, bariatric surgery can also be indicated for the control of weight-related metabolic comorbidities, such as T2DM, when it is called metabolic surgery^41^. It is estimated that T2DM currently affects about 360 million adults, and of these, almost half are obese, and bariatric and metabolic surgery are the most effective treatment to control these conditions, reducing or even preventing associated complications^41,42^.

This study found a statistically significant relationship between obese patients with diabetes and the type of bariatric surgery performed, with RYGB being the procedure that was most often performed in these cases. Recent reports have shown that RYGB has a relative superiority in weight loss and long-term glycemic control^42,43^. However, there is no consensus in the literature and SG is also indicated for the metabolic control of diabetes in obese patients. According to Mc Tigue et al.^44^, RYGB ensures a longer time to remission of diabetes than SG, with recurrence rates of uncontrolled glycemia for RYGB and SG of 33.1% and 41.6%, respectively.

The authors highlight some limitations in the study. The main one is the retrospective nature of the analysis. In addition, the sample is composed only of patients linked to two medium complexity health centers that undergo bariatric surgery and does not cover all the services that provide this service in the region. In addition, the research used data obtained from medical records, in which some did not follow a specific standardization, which may compromise the obtaining of some relevant data for the research objectives and, consequently, its analysis.

## CONCLUSION

The clinical-epidemiological profile of patients who underwent bariatric surgery in the southern region of Maranhão is compatible with the national scenario. The findings reflect an important perspective of the local reality and may serve as a pilot for the future development of larger studies and Health Policies, considering the local particularities observed. The characterization and analysis of the clinical-epidemiological profile of patients undergoing bariatric and metabolic surgery is relevant for the reduction of obesity and weight-related conditions, for the organization and expansion of access to this service, and for the future planning of new actions to improve the quality and expectancy of life in this group.
